# MIRSIG position paper: the use of image registration and fusion algorithms in radiotherapy

**DOI:** 10.1007/s13246-022-01125-3

**Published:** 2022-05-06

**Authors:** Nicholas Lowther, Rob Louwe, Johnson Yuen, Nicholas Hardcastle, Adam Yeo, Michael Jameson, the Medical Image and Registration Special Interest Group (MIRSIG) of the ACPSEM

**Affiliations:** 1 Department of Radiation Oncology, Wellington Blood and Cancer Centre, Wellington, New Zealand; 2 Holland Proton Therapy Centre, Delft, Netherlands; 3 St George Hospital Cancer Care Centre, Kogarah, New South Wales 2217 Australia; 4 South Western Clinical School, University of New South Wales, Sydney, Australia; 5 Ingham Institute for Applied Medical Research, Sydney, NSW Australia; 6 Physical Sciences, Peter MacCallum Cancer Centre, Melbourne, VIC Australia; 7 Centre for Medical Radiation Physics, University of Wollongong, Wollongong, NSW Australia; 8 The Sir Peter MacCallum Department of Oncology, The University of Melbourne, Melbourne, VIC Australia; 9 School of Applied Sciences, RMIT University, Melbourne, VIC Australia; 10 GenesisCare, Sydney, NSW 2015 Australia; 11 St Vincent’s Clinical School, University of New South Wales, Sydney, Australia

**Keywords:** Deformable image registration, Image fusion, Dose warping, Quality assurance, Patient-specific verification

## Abstract

The report of the American Association of Physicists in Medicine (AAPM) Task Group No. 132 published in 2017 reviewed rigid image registration and deformable image registration (DIR) approaches and solutions to provide recommendations for quality assurance and quality control of clinical image registration and fusion techniques in radiotherapy. However, that report did not include the use of DIR for advanced applications such as dose warping or warping of other matrices of interest. Considering that DIR warping tools are now readily available, discussions were hosted by the Medical Image Registration Special Interest Group (MIRSIG) of the Australasian College of Physical Scientists & Engineers in Medicine in 2018 to form a consensus on best practice guidelines. This position statement authored by MIRSIG endorses the recommendations of the report of AAPM task group 132 and expands on the best practice advice from the ‘Deforming to Best Practice’ MIRSIG publication to provide guidelines on the use of DIR for advanced applications.

**Supplementary Information:**

The online version contains supplementary material available at 10.1007/s13246-022-01125-3.

## Introduction

Registration and fusion of medical images [1, 2] has become an integral component of a wide range of procedures within radiation oncology which are increasingly being used to inform and drive clinical decisions. Target and/or normal tissue delineation, image-guided treatment, response assessment, re-planning and plan adaptation are example procedures in a patient’s treatment workflow which are now generally underpinned by image registration (IR) and fusion processes. These IR and fusion processes typically manipulate multimodal, anatomical atlas and/or time-series image data and their use in radiotherapy is expected to increase in the near future [3–8]. However it should be recognised that IR is an imperfect process and spatial registration uncertainties may still be present after the IR and/or fusion has been performed. These uncertainties can be caused by sub-optimal image quality, inappropriate use of the registration algorithm’s parameters, use of registration algorithms without consideration of their limitations, or incorrect interpretation of the registration results. In this context, guidance to assist the validation, commissioning and clinical integration of IR and fusion techniques is warranted.

The report of the American Association of Physicists in Medicine (AAPM) Radiation Therapy Committee Task Group No. 132 [9] published in 2017 reviewed rigid image registration (RIR) and deformable image registration (DIR) approaches and solutions to provide recommendations for quality assurance (QA) and quality control (QC) of clinical IR and fusion techniques in radiotherapy. However, the use of DIR for the advanced applications of dose warping or warping of other matrices such as standardised uptake values (SUVs) in positron emission tomography (PET), ventilation maps and distortion corrections in magnetic resonance imaging (MRI) were outside the scope of the report [9]. In part to address this need, a recent publication [10] authored by the Society for Medical Image Registration and Fusion (or SMIRF), now known as the Medical Image Registration Special Interest Group (MIRSIG) of the Australasian College of Physical Scientists & Engineers in Medicine (ACPSEM) aimed to communicate limitations and provide best practice advice to departments in the Australia and New Zealand setting that have implemented DIR, or that are planning to implement DIR in the near future. The recently established MIRSIG is a multidisciplinary special interest group that aims to: (1) provide a strong and unified driving force for the management of medical IR in Australasia; and (2) provide professional standards and solutions for safe and effective use of medical IR for the benefit of the public.

This position statement authored by MIRSIG and consulted experts endorses the recommendations of the report of AAPM task group 132 [9] and expands upon the best practice advice from the ‘Deforming to Best Practice’ MIRSIG publication [10] to provide guidelines on the use of DIR for advanced applications. While this position statement has been prepared by radiation oncology professionals for use in radiotherapy departments, it may provide guidance for professionals working in radiology, nuclear medicine and radiopharmaceutical science.

## Terminology

MIRSIG acknowledges the need to clearly identify and consistently use terminology in the context of IR and fusion evaluation, as was highlighted in the report of TG-132 [9]. This position statement uses the following terminology which is consistent with that report and the recent MIRSIG publication [10]:

- Moving dataset: The dataset that is being transformed or deformed to match another image.
- Stationary dataset: The dataset that another image is being registered to.
- Image registration (IR): The process of determining the geometric transformation that relates identical points in two image series: a moving dataset and a stationary dataset.
- Image fusion: The combined display of the mapped data from the moving dataset with the stationary dataset.
- Rigid image registration (RIR): A registration where the transformation preserves the distance between all points in the image. A rigid registration can include translation in all directions as well as rotations in all directions.
- Deformable image registration (DIR): A registration transformation that does not preserve the distance between all points in the image. The number of degrees of freedom can be as large as three times the number of voxels in the source dataset (e.g., a unique displacement vector for every voxel in the source dataset).
- Deformation vector field (DVF): A transform describing the vector needed for each voxel to generate a warped image.
- Target registration error (TRE): Point-based accuracy metric using implanted or naturally occurring landmarks visualised on a pair of images [11].
- Mean distance to agreement (MDA): Mean surface distance between two contours on registered images [12].
- Dice similarity coefficient (DSC): Volumetric overlap of two contours on registered images [13].
- Jacobian determinant: Volume expansion or contraction resulting from a DIR [14].
- Consistency: Independence of the transformation result to the direction of the registration (image A to image B or image B to image A) [15].
- Transitivity: Independence of the transformation result to the registration scheme with more than two images (image A to image C or image A to image B to image C) [16].
- Validation: The evaluation of the overall process and toolset to ensure that accurate image registration can be performed on a consistent basis for the intended use.
- Verification: The process of confirming that the accuracy of a specific image registration is acceptable for the intended use.
- Quality Assurance (QA): The procedures and process followed to ensure maintenance of quality in each image registration.
- Commissioning: The process of validating the IR system, verifying example patient cases, generating appropriate documentation and providing training to users of the IR system.
- Radiation oncology professional trained in DIR: A radiation oncology medical physicist (ROMP) [17, 18], radiation therapist (RT), radiation oncologist (RO) or similar that has attended and actively participated in workshops and/or training courses provided by professional organisations on DIR relevant to radiotherapy.

## MIRSIG position on tools for validation, verification and quality assurance of image registration

The majority of commercially available IR and fusion software packages tailored for radiotherapy provide some quantitative and qualitative evaluation tools which can be used for validation, verification and QA. These tools should be used specifically for QA at treatment planning and re-planning, when commissioning an IR system and for verification at treatment delivery. MIRSIG endorses the use of the following tools either described in the report of TG-132 [9] or used locally to qualitatively evaluate the IR:

- Split screen
- Checkerboard
- Image overlay
- Difference image
- Contour/structure mapping displays
- Cine images (assessment of 4D deformation)

Assessing the registration of a pre-treatment cone beam computed tomography (CBCT) image to the planning CT image with the image overlay tool is a classic example of qualitative IR evaluation. An example use of the contour/structure mapping display tool is the visual evaluation of accuracy of a structure (e.g. the gross tumour volume) contoured on an MRI image but displayed on the registered planning CT image.

MIRSIG endorses the use of the quantitative tools and associated tolerances listed in Table 1 for IR processes. The need to assess the consistency and, as necessary, the transitivity of an IR is acknowledged. However, it should be noted that an IR system within tolerance for consistency demonstrates the system is inverse consistent but does not necessarily ensure that a specific IR is clinically acceptable or spatially accurate. More in-depth descriptions of spatial consistency evaluation metrics are provided in the literature [19, 20] and their use may provide a more thorough means of evaluation compared to the tools listed in Table 1. The specific qualitative and quantitative tools used for validation, verification and QA of the IR and/or fusion will be dependent on the specific radiotherapy procedure (e.g. structure delineation, online adaption based on pre-treatment verification imaging, response assessment, etc.). Examples of specific IR and fusion processes which could be evaluated with each tool are listed in Table 1 with references to the literature where they have been utilised in the context of radiotherapy.

**Table 1:** Quantitative tools, their associated tolerances and example image registration (IR) and fusion processes to evaluate. Adapted from the report of AAPM task group 132 [9]

Evaluation tool	Tolerance	Example IR and fusion processes to evaluate
Target registration error (TRE)	Maximum voxel dimension (~ 2–3 mm)	• The result of a rigid or deformable IR prior to its use for structure delineation [34, 35]. E.g. the rigid IR of an MRI image to the planning CT image • Deformed image accuracy [36, 37]
Mean distance to agreement (MDA)	Within the contouring uncertainty of the structure or maximum voxel dimension (~ 2–3 mm)	• Structure treatment response/assessment of contour propagation (on time-series data) [38] • Assessment of intra-/inter-user contouring uncertainty [39]
Dice similarity coefficient (DSC)	Within the contouring uncertainty of the structure (~ 0.80–0.90^a^) [40]	• Structure treatment response/assessment of contour propagation (on time-series data) [38] • Assessment of intra-/inter-user contouring uncertainty [39, 41]
Jacobian determinant	No negative values, values deviating from 1 as expected from clinical scenario (0–1 for structures expected to reduce in volume, greater than 1 for structures expected to expand in volume)	• DVF used for contour propagation [22] • DVF used to generate a deformed image [22]
Inverse consistency	Maximum voxel dimension (~ 2–3 mm)	• DVF used to generate a deformed image [22]

^a^DSC calculations are dependent on the volume of the structure, therefore very large or very small structures may have different expected DSC values for contour uncertainty

Not all evaluation tools are suited for the more advanced applications of DIR where the result of the registration is used to deform a dose or other matrix (e.g. PET SUV, ventilation maps, MRI distortion corrections). For example, accurate DIR-facilitated warped doses require accurate tissue-to-tissue mapping and not all tools evaluate all voxels within structures. In contrast for contour propagation, DVF errors occurring in a structure’s sub-volume are inconsequential if the structure’s boundaries are accurately mapped. For the evaluation of DIR-facilitated dose and other matrix warping, MIRSIG encourages the use of the following quantitative evaluation tools *in addition* to those listed in Table 1:

- DVF histograms
- Jacobian maps
- Transitivity error (TE)
- Harmonic energy (HE)

Descriptions and examples of these tools evaluating DIR in the context of radiotherapy can be found in the literature [21–24].

Low image contrast, image distortion, noise, artefacts, algorithm restrictions/limitations and large anatomical changes are examples of factors that can cause DVF errors [25]. Specific to DIR-facilitated dose warping (although comparable to SUV warping of PET images), erroneous DVFs which are applied to a dose distribution will cause errors in the warped dose distributions. While the impact of DIR error on the accuracy of dose warping is not yet fully understood [25], a number of studies have investigated the impact of DVF errors on warped dose distributions [26–32]. In general, these studies have demonstrated that the spatial distribution of uncertainties in DIR-facilitated warped doses are highly heterogeneous. In addition, it has been demonstrated that highly accurate DVFs are needed in regions of large-dose gradients [26–32].

It is the MIRSIG position that radiotherapy IR and fusion software packages should facilitate import and export of the registration transformation matrices or DVFs to allow independent validation. MIRSIG also endorses the goals of the Integrating the Healthcare Enterprise (IHE) Radiation Oncology Technical Framework Supplement—Deformable Registration in Radiation Oncology (DRRO) [33] of having full inter-operability of DIR results through the Digital Imaging and Communications in Medicine (DICOM) standard.

## MIRSIG position on commissioning and validating image registration software

Physical phantom end-to-end tests, digital phantom tests and clinical data tests are recommended in the report of TG-132 [9] to commission and validate IR and fusion software systems, which MIRSIG endorses. Physical phantoms may facilitate system end-to-end tests to ensure accurate and consistent data representation, image transfer, integrity between image acquisition devices, IR systems and systems that use the IR results [9]. Digital phantoms allow comprehensive testing of the IR accuracy against a known ground truth (note this may also be performed with physical phantoms). Clinical data tests that include clinically observed deformations provide final validation of the IR and fusion system’s accuracy [9]. MIRSIG endorses the evaluations outlined in Table IV from the report of TG-132 [9] for commissioning, annual QA and upon upgrade of an IR and fusion system.

Digital phantoms can be generated in house or purchased from vendors and either can be used to perform the evaluation tests outlined in Table IV [9] that require digital phantoms. It is worth noting however that incompatibilities have been reported between digital phantoms and commercial systems [42]. MIRSIG endorses the digital phantom datasets of the report of TG-132 [9] (available from https://www.aapm.org/pubs/reports/report132.asp) and additional open-source datasets for DIR validation, which can be found in Supplementary Material A. Please note that the majority of the linked open-source datasets contain images only and have limited ability for ground-truth evaluation (i.e. a lack of inclusion of landmarks, contours, deformed doses, known DVFs, etc.). However, efforts are on-going for their inclusion.

Physical and digital phantoms as well as real patient data have been utilised in attempts to validate DIR-facilitated dose warping [32, 43–49]. While such studies are helping to elucidate the dosimetric uncertainties of DIR-facilitated dose warping, these approaches do not necessarily expose all possible limitations associated with DIR-facilitated dose reconstruction. DIR algorithms do generally assume that mass is conserved and are therefore not able to correctly replicate volume changes. Consequently, DIR algorithms can violate the principle that energy should be conserved and as a result the suitability of DIR for accurate and precise dose warping and subsequent accumulation has been debated in the literature [44, 50–53]. Given the fact that it is non-trivial to handle such limitations with the currently available tools, MIRSIG recommends characterising DIR algorithms at least to some extent for clinically relevant scenarios. This may be achieved by comparing the DVFs of mass conserving and non-mass conserving registration scenarios using the qualitative and quantitative evaluation tools described in section ‘MIRSIG position on tools for validation, verification and quality assurance of image registration’. Mass conserving registration scenarios may include the IR between 3D volumes of a 4D dataset or the IR of two images acquired on the same day with different setup positions (e.g. neck tilt). Non-mass conserving registration scenarios may include the IR of pre-treatment images (e.g. CBCT) to the planning CT which can show anatomical changes such as organ emptying/filling, tumour shrinkage/growth, weight loss or the presence/absence of immobilisation devices.

## MIRSIG position on patient-specific registration verification during clinical practice

MIRSIG advocates the evaluations outlined in Table 2 for patient-specific verification of IR and fusion, which are adapted from the report of TG-132 [9] and MIRSIG publication [10]. Clear and consistent communication regarding IR and fusion is imperative considering the results will generally be used in a multidisciplinary manner, and for multiple procedures in a patient’s treatment workflow. MIRSIG endorses the use of the request and report forms found in Appendix B of the report of TG-132 [9] for straightforward applications of RIR and DIR. For example, when registering multimodality images to aid structure contouring or for simple assessments of treatment response. Note that the definitions of the phrases used in these forms can be found in Table VII of the report of TG-132 [9]. In cases where DIR is used in a more complex manner such as warping dose or other matrices, the request and report forms of the report of TG-132 [9] may not be sufficient. In these cases, the use of an extensive application-specific IR request and report form is endorsed. An example of such an IR request and report form has been developed by MIRSIG and can be found in Supplementary Material B.

**Table 2:** Evaluations and their criteria adapted from the report of AAPM task group 132 and Medical Image Registration Special Interest Group (MIRSIG) publication [10] for patient-specific IR verification

Use case	Evaluation	Criteria
Each patient	Data transfer	Exact
	Patient orientation	Image data matches specified orientation (superior/inferior, anterior/posterior, left/right)
	Image size	Qualitative—no observable distortions, correct aspect ratio
	Data integrity and import	User defined per TG53 recommendations
	Contour propagation	Visual confirmation that contours are within 1–2 voxels of visible boundaries of anatomy
	Rigid registration accuracy	Confirmation that the observed deviations of the relevant anatomical boundaries in the registered images are consistent with the magnitude that was used in the margin calculations [54, 55]
	Deformable registration accuracy	Confirmation that the observed deviations of the relevant anatomical boundaries in the registered images are consistent with the magnitude that was used in the margin calculations [54, 55]. Evaluate the reasonableness of the deformation vector field and perform a quantitative evaluation
	DIR-facilitated dose warping accuracy [10] (*in addition to the evaluations listed above*)	• IR request and report forms • DIR reviewed by appropriately trained radiation oncology professional* • The specific quantitative tools: DVF histograms, Jacobian maps, inverse consistency and harmonic energy. *In addition* to those tools listed in Table 1 • New data sets resampled and named according to department rules • Independent check of correct data sets and processes used, and correct weighting applied to each input dose

*As defined in the ‘Terminology’ section. A ROMP, RT, RO or similar that has attended and actively participated in workshops and/or training courses provided by professional organisations on DIR relevant to radiotherapy

While current tools and workflows for DIR-facilitated dose and other matrix warping are still in their infancy with regards to clinical use [25], suggested patient-specific verification has been described by MIRSIG [10] and is included in Table 2. Patient-specific verification should be approved by a radiation oncology professional trained in DIR (see ‘Terminology’ section for definition).

## MIRSIG position on clinical integration of registration techniques in treatment planning and delivery

MIRSIG advocates the following recommendations which are combined from the report of TG-132 [9], the MIRSIG publication [10] and local consensus to ensure an efficient and safe clinical integration of IR and fusion systems:

1. Clear guidelines, rules and training are provided to the personnel performing the IR and/or fusion on what results to accept or not, and when to escalate if necessary.
2. An efficient, patient-specific verification is performed for each IR prior to its use, as appropriate. For example, a qualitative assessment of the registration of a pre-treatment CBCT image to the planning CT image using only the image overlay tool is likely sufficient. In comparison, the majority of the quantitative tools listed in section ‘MIRSIG position on tools for validation, verification and quality assurance of image registration’ are likely to be needed to verify a patient’s accumulated dose established with DIR-facilitated dose warping.
3. Registration accuracy is assessed at a frequency to minimise the effect of errors without prohibiting clinical flow.
4. Clear identification of the accuracy of the registration is provided to the consumer (e.g. an RO who receives a resultant IR prior to contouring) of the image fusion so they are fully aware of and can account for any uncertainties.
5. Policies and procedures are in place for data management. This is useful for tracking data, performing tasks in the correct order and deciding which workspace each task will be performed in.
6. Sufficient resources are provided for IR processes, including its commissioning and QA.
7. Workflows implementing IR should be assessed to prove net clinical gain (e.g. demonstrable time and accuracy gains when manually reviewing/editing DIR-propagated/atlas-based contours compared to manually delineating contours from scratch [41]).
8. Clear rules regarding algorithm limitations or restrictions are provided to performers and consumers of IR and/or fusion systems.

## Summary of clinical recommendations

MIRSIG endorses the clinical recommendations for IR and fusion systems which were summarised in the report of TG-132 [9] and detailed in the MIRSIG publication [10]. These have been slightly adapted as follows:

1. All users should understand the basic IR techniques and methods of visualising image fusion.
2. All users should understand the basic components of the registration algorithm used clinically to ensure its proper use.
3. If the IR is performed on a stand-alone system, end-to-end tests should be performed with a physical phantom for validation. Note that the stand-alone IR system must support exportation of intermediate results.
4. Perform comprehensive commissioning of IR using the linked digital phantom data (or similar data) as well as clinical data from the user’s institution
5. Estimation of registration error should be assessed using a combination of the quantitative and qualitative evaluation tools described in section ‘MIRSIG position on tools for validation, verification and quality assurance of image registration’. Larger uncertainties should be included in the margin calculations [54, 55].
6. Develop a request and report system to ensure clear communication and documentation between all users of IR.
7. Establish a patient-specific QA practice for efficient evaluation of IR results.
8. Appropriate training and education is given to staff performing the IR and staff integrating the results of the IR in patients’ clinical management.
9. Understand the benefits and risks of IR using a risk-based framework [56] for each clinical application and anatomical site which are also department specific.

## Supplementary Information

Below is the link to the electronic supplementary material.

Supplementary file1 (PDF 839 kb)

Supplementary file2 (XLSX 472 kb)

## References

[1] Hill DLG, Batchelor PG, Holden M, Hawkes DJ (2001). Medical image registration. Phys Med Biol.

[2] Kessler ML (2006). Image registration and data fusion in radiation therapy. Br J Radiol.

[3] Yuen J, Barber J, Ralston A (2020). An international survey on the clinical use of rigid and deformable image registration in radiotherapy. J Appl Clin Med Phys.

[4] Kisling KD, Ger RB, Netherton TJ (2018). A snapshot of medical physics practice patterns. J Appl Clin Med Phys.

[5] Batumalai V, Holloway LC, Kumar S (2017). Survey of image-guided radiotherapy use in Australia. J Med Imaging Radiat Oncol.

[6] Viergever MA, Maintz JBA, Klein S (2016). A survey of medical image registration—under review. Med Image Anal.

[7] Hussein M, Akintonde A, McClelland J (2021). Clinical use, challenges, and barriers to implementation of deformable image registration in radiotherapy—the need for guidance and QA tools. Br J Radiol.

[8] Kadoya N, Kito S, Kurooka M (2019). Factual survey of the clinical use of deformable image registration software for radiotherapy in Japan. J Radiat Res.

[9] Brock KK, Mutic S, McNutt TR (2017). Use of image registration and fusion algorithms and techniques in radiotherapy: report of the AAPM Radiation Therapy Committee Task Group No. 132: report. Med Phys.

[10] Barber J, Yuen J, Jameson M (2020). Deforming to best practice: key considerations for deformable image registration in radiotherapy. J Med Radiat Sci.

[11] Fitzpatrick JM, West JB, Maurer CR (1998). Predicting error in rigid-body point-based registration. IEEE Trans Med Imaging.

[12] Chalana V, Kim Y (1997). A methodology for evaluation of boundary detection algorithms on medical images. IEEE Trans Med Imaging.

[13] Dice L (1945). Measures of the amount of ecologic association between species. Ecology.

[14] Leow AD, Yanovsky I, Chiang MC (2007). Statistical properties of Jacobian maps and the realization of unbiased large-deformation nonlinear image registration. IEEE Trans Med Imaging.

[15] Christensen GE, Johnson HJ (2001). Consistent image registration. IEEE Trans Med Imaging.

[16] Christensen GE, Johnson HJ (2003). Invertibility and transitivity analysis for nonrigid image registration. J Electron Imaging.

[17] Australasian College of Physical Scientists & Engineers in Medicine (2014) ACPSEM position on the roles and responsibilities of the qualified medical physicist. Version 2.3

[18] Australasian College of Physical Scientists & Engineers in Medicine (2018) ACPSEM position statement: the role of physicists, scientists and engineers in medicine in Australasia

[19] Bender ET, Tomé WA (2009). The utilization of consistency metrics for error analysis in deformable image registration. Phys Med Biol.

[20] Saleh ZH, Apte AP, Sharp GC (2014). The distance discordance metric—a novel approach to quantifying spatial uncertainties in intra- and inter-patient deformable image registration. Phys Med Biol.

[21] Paganelli C, Meschini G, Molinelli S (2018). Patient-specific validation of deformable image registration in radiation therapy: overview and caveats. Med Phys.

[22] Varadhan R, Karangelis G, Krishnan K (2013). A framework for deformable image registration validation in radiotherapy clinical applications. J Appl Clin Med Phys.

[23] Kierkels RJ, den Otter LA, Korevaar EW (2018). An automated, quantitative, and case-specific evaluation of deformable image registration in computed tomography images. Phys Med Biol.

[24] Bender ET, Hardcastle N, Tomé WA (2012). On the dosimetric effect and reduction of inverse consistency and transitivity errors in deformable image registration for dose accumulation. Med Phys.

[25] Chetty IJ, Rosu-Bubulac M (2019). Deformable registration for dose accumulation. Semin Radiat Oncol.

[26] Hub M, Thieke C, Kessler ML, Karger CP (2012). A stochastic approach to estimate the uncertainty of dose mapping caused by uncertainties in b-spline registration. Med Phys.

[27] Murphy MJ, Salguero FJ, Siebers JV (2012). A method to estimate the effect of deformable image registration uncertainties on daily dose mapping. Med Phys.

[28] Yan C, Hugo G, Salguero FJ (2012). A method to evaluate dose errors introduced by dose mapping processes for mass conserving deformations. Med Phys.

[29] Samavati N, Velec M, Brock KK (2016). Effect of deformable registration uncertainty on lung SBRT dose accumulation. Med Phys.

[30] Salguero FJ, Saleh-Sayah NK, Yan C, Siebers JV (2011). Estimation of three-dimensional intrinsic dosimetric uncertainties resulting from using deformable image registration for dose mapping. Med Phys.

[31] Tilly D, Tilly N, Ahnesjö A (2013). Dose mapping sensitivity to deformable registration uncertainties in fractionated radiotherapy—applied to prostate proton treatments. BMC Med Phys.

[32] Lowther NJ, Marsh SH, Louwe RJW (2020). Quantifying the dose accumulation uncertainty after deformable image registration in head-and-neck radiotherapy. Radiother Oncol.

[33] IHE Radiation Oncology Technical Committee (2021) IHE radiation oncology technical framework supplement—deformable registration in radiation oncology (DRRO). Revision 1.0. https://www.ihe.net/resources/public_comment/#radiationoncology

[34] Dean CJ, Sykes JR, Cooper RA (2012). An evaluation of four CT-MRI co-registration techniques for radiotherapy treatment planning of prone rectal cancer patients. Br J Radiol.

[35] Cattaneo G, Reni M, Rizzo G (2005). Target delineation in post-operative radiotherapy of brain gliomas: interobserver variability and impact of image registration of MR (pre-operative) images on treatment planning CT scans. Radiother Oncol.

[36] Mencarelli A, van Beek S, van Kranen SR (2012). Validation of deformable registration in head and neck cancer using analysis of variance. Med Phys.

[37] Hou J, Guerrero M, Chen W, D’Souza WD (2011). Deformable planning CT to cone-beam CT image registration in head-and-neck cancer. Med Phys.

[38] Hardcastle N, Tomé WA, Cannon DM (2012). A multi-institution evaluation of deformable image registration algorithms for automatic organ delineation in adaptive head and neck radiotherapy. Radiat Oncol.

[39] Vinod SK, Jameson MG, Min M, Holloway LC (2016). Uncertainties in volume delineation in radiation oncology: a systematic review and recommendations for future studies. Radiother Oncol.

[40] Sharp G, Fritscher KD, Pekar V (2014). Vision 20/20: perspectives on automated image segmentation for radiotherapy. Med Phys.

[41] Ramadaan IS, Peick K, Hamilton DA (2015). Validation of Varian’s SmartAdapt® deformable image registration algorithm for clinical application. Radiat Oncol.

[42] Rong Y, Rosu-Bubulac M, Benedict SH, Cui Y (2021). Rigid and deformable image registration for radiation therapy: a self-study evaluation guide for NRG Oncology clinical trial participation. Pract Radiat Oncol.

[43] Bohoudi O, Lagerwaard FJ, Bruynzeel AME (2019). End-to-end empirical validation of dose accumulation in MRI-guided adaptive radiotherapy for prostate cancer using an anthropomorphic deformable pelvis phantom. Radiother Oncol.

[44] Yeo UJ, Taylor ML, Supple JR (2012). Is it sensible to “deform” dose 3D experimental validation of dose-warping. Med Phys.

[45] Veiga C, Lourenço AM, Mouinuddin S (2015). Toward adaptive radiotherapy for head and neck patients: uncertainties in dose warping due to the choice of deformable registration algorithm. Med Phys.

[46] Nassef M, Simon A, Cazoulat G (2016). Quantification of dose uncertainties in cumulated dose estimation compared to planned dose in prostate IMRT. Radiother Oncol.

[47] Thor M, Andersen ES, Petersen JBB (2014). Evaluation of an application for intensity-based deformable image registration and dose accumulation in radiotherapy. Acta Oncol (Madr).

[48] Graves YJ, Smith AA, McIlvena D (2015). A deformable head and neck phantom with in-vivo dosimetry for adaptive radiotherapy quality assurance. Med Phys.

[49] Niu CJ, Foltz WD, Velec M (2012). A novel technique to enable experimental validation of deformable dose accumulation. Med Phys.

[50] Schultheiss TE, Tome WA, Orton CG (2012). Point/counterpoint: it is not appropriate to “deform” dose along with deformable image registration in adaptive radiotherapy. Med Phys.

[51] Zhong H, Chetty IJ (2017). Caution must be exercised when performing deformable dose accumulation for tumors undergoing mass changes during fractionated radiation therapy. Int J Radiat Oncol Biol Phys.

[52] Hugo GD, Dial C, Siebers JV (2017). In regard to Zhong and Chetty. Int J Radiat Oncol.

[53] Taylor ML, Yeo UJ, Kron T et al (2013) Comment on “It is not appropriate to ‘deform’ dose along with deformable image registration in adaptive radiotherapy” [Med. Phys. 39, 6531–6533 (2012)]. Med Phys. 10.1118/1.4771962 10.1118/1.4771962 23298128

[54] Van Herk M (2004). Errors and margins in radiotherapy. Semin Radiat Oncol.

[55] Van Herk M, Remeijer P, Rasch C, Lebesque JV (2000). The probability of correct target dosage: dose-population histograms for deriving treatment margins in radiotherapy. Int J Radiat Oncol Biol Phys.

[56] Huq MS, Fraass BA, Dunscombe PB (2016). The report of Task Group 100 of the AAPM: application of risk analysis methods to radiation therapy quality management. Med Phys.

