# Supplementary material for: MIRSIG position paper: the use of image registration and fusion algorithms in radiotherapy
Source: Phys Eng Sci Med. 2022 May 6;45(2):421–8. doi: 10.1007/s13246-022-01125-3 (PMC9239966; doi:10.1007/s13246-022-01125-3)
Supplement: Supplementary file 1 — Supplementary file1 (PDF 839 kb) [file 13246_2022_1125_MOESM1_ESM.pdf]

ACPSEM Australasian College of Physical Scientists & Engineers in Medicine

MIRSIG

Open Source Datasets
For Deformable Image Registration Validation V1.2

Version	Date	Data recommended-Initials	Change Request	Change Complete - Initial
1.0	17/10/2019	Caitlin A	Initial draft made and reviewed by Nick H and Adam Y	Caitlin A
1.0	25/03/2020	Adam Y	Sent to MIRSIG group for comments	Adam Y
1.1	18/08/2020	Shrikant D<br>Alison G	Added 4D-CBCT and 4D-FBCT from TCIA<br>Added H&N (Pukala et al), previously excluded by AY	Adam Y
1.2	18/9/20	Johnson Y	Formatting/Logos, added notes on additional datasets for TCIA, added TG132 datas	

Preamble

There is no conflict of interest in collecting open-source datasets

To generate an open-source data registry is one of on-going projects under Medical Image Registration Special Interest Group (MIRSIG), ACPSEM. The idea is to collect as many open source datasets as possible from experts in medical imaging and radiation oncology community with a primary aim of using it to validate deformable image registration (DIR) systems plus a secondary aim to identify any gap as per clinical need (e.g. body sites). MIRSIG may continue to the list.

We hope that MIRSIG can continue to contribute to the list and improve it in future, by providing landmarks or contours on the identified datasets if needed, which can serve as common resources for DIR evaluation within the community.

A list of open-source datasets

1. Continuous Registration Challenge (CRC)

- Has 7 public data sets and 1 private
- Looking at brain and lung registrations
- POPI
- DIRLAB
- Empire
- LONI Probabilistic Brain Atlas (LPBA40)
- Internet Brain Segmentation Repository (IBSR)
- CUMC12
- MGH10

https://continuousregistration.grand-challenge.org/data/

2. POPI

- Point-validated Pixel-based breathing thorax model
- 1 x 4DCT with 10 3DCT images
- 40 landmarks for each 3DCT
- DVFs obtained using 2 registration methods between two breathing phases
- Described in: **J. Vandemeulebroucke, D. Sarrut, and P. Clarysse**
- ***The POPI-model, a point-validated pixel-based breathing thorax model.***
- **In *Proceeding of the XVth ICCR Conference*, Toronto, Canada. 2007**

https://www.creatis.insa-lyon.fr/rio/popi-model_original_page

3. DIRLAB

- Requires registration to access open source datasets
- 10 thoracic 4DCT datasets
- Images in extreme phases annotated with 300 landmarks
- *Castillo R, Castillo E, Guerra R, Johnson VE, McPhail T, Garg AK, Guerrero T. 2009. A framework for evaluation of deformable image registration spatial accuracy using large landmark point sets. Phys Med Biol 54 1849-1870*
- *Castillo E, Castillo R, Martinez J, Shenoy M, Guerrero T. 2009. Four-dimensional deformable image registration using trajectory modeling. Phys Med Biol 55 305-327*

https://www.dir-lab.com/Downloads.html

4. Empire

- Set of 30 thoracic scan pairs
- *Murphy et al., "Evaluation of registration methods on thoracic CT: the EMPIRE10 challenge.", IEEE Trans Med Imaging. 2011 Nov;30(11):1901-20*

https://empire10.grand-challenge.org/download/

5. LONI Probabilistic Brain Atlas (LPBA40)

- Not really a registration dataset but includes different variants
- Whole head MRI of 40 volunteers with manual delineations of 56 brain structures
- *Shattuck DW, Mirza M, Adisetiyo V, Hojatkashani C, Salamon G, Narr KL, Poldrack RA, Bilder RM, Toga AW, Construction of a 3D Probabilistic Atlas of Human Cortical Structures, NeuroImage (2007), doi: 10.1016/j.neuroimage.2007.09.031*

http://www.loni.usc.edu/research/atlas_downloads

6. Internet Brain Segmentation Repository (IBSR)

- brain
- Manually-guided segmentation results (shown as green outlines) are provided for some data sets along with the corresponding MR images
- The segmentation or "outline" files are the result of semi-automated segmentation techniques which require many hours of effort by a trained expert
- Used for comparing automated segmentation results
- To obtain this dataset, download "IBSR_v2.0_nifti_stripped.thz" from https://www.nitrc.org/projects/ibsr/.

7. CUMC12

- brain
- 18 images acquired at the Columbia University Medical Center
- Segmented and manually labelled
- 128 labelled regions

https://www.synapse.org/#!Synapse:syn3207203

8. MGH10

- brain
- 10 subjects scanned at the MGH/MIT/HMS Athinoula A. Martinos Center for Biomedical Imaging
- The data is inhomogeneity-corrected, affine-registered to the MNI152 template (Evans et al., 1992), and segmented using SPM2 software (Friston et al., 1995) into 74 regions according to the labeling protocol by Tourville and Guenther, 2003.

https://www.synapse.org/#!Synapse:syn3207203

9. Retrospective Image Registration Evaluation Project

- The project is designed to compare retrospective CT-MR and PET-MR registration techniques used by a number of groups.
- Has training data with transformations and test data

https://www.insight-journal.org/rire/download.php

10. Annotated MRI and ultrasound volume images of the prostate

- MRI and ultrasound prostate images
- Each patient has MRI and US image of the prostate
- Contoured prostate, and included some fiducials

https://zenodo.org/record/16396#.XdcvL3txWUk

11. Volumetric Non-Rigid Registration for MRI-guided Brain Tumor Surgery

- Brain

http://2014.ncigt.org/publications/item/view/541

12. MR-guided Prostate Biopsy pre- and intra-procedural MRI Registration Evaluation Dataset

- Prostate
- The dataset contains MRI volumes and additional data collected for 10 patients that underwent MR-guided prostate biopsy.
- These data were used for evaluation of the non-rigid registration method presented in the following publication.
- For each patient, the following elements are provided: (1) pre-procedural and intra-procedural T2w MRI; (2) approximate segmentation of the prostate gland by non-expert used for the initialization of the registration method, for each image volume; (3) segmentation of the central gland and peripheral zone by two abdominal radiologists, for each image volume; (4) points identifying the corresponding patient-specific anatomical landmarks in the pre- and intra-procedural volumes, for each patient together with the segmentations and anatomical landmarks

http://2014.ncigt.org/publications/item/view/2147

13. The Slicer Registration Case Library

- No longer maintained
- Wide range of cases including brain, breast, liver, prostate, and kidney

https://www.na-mic.org/wiki/Projects:RegistrationDocumentation:UseCaseInventory

14. FIRE: Fundus Image Registration Dataset

- The dataset consists of 129 retinal images forming 134 image pairs.
- Annotated with ground truth data

https://www.ics.forth.gr/cvrl/fire/

15. CQ500 Dataset

- 491 individual CT scans and the corresponding radiologists' reads

http://headctstudy.qure.ai/#dataset

16. Multiresolution image registration for multimodal brain images and fusion for better neurosurgical planning

- CT and MRI datasets for 8 patients

https://data.mendeley.com/datasets/2rh8mftdnf/1

17. The Cancer Imaging Archive (TCIA): 4D-Lung dataset

- 4D-fan beam (4D-FBCT) and 4D-cone beam CT (4D-CBCT) from 20 locally-advanced, non-small cell lung cancer patients.
- 10 bins for both 4D-FBCT and 4D-CBCT
- Data collection and analysis was supported by NIH P01CA116602
- *S. Balik et al. 2013 Evaluation of 4-Dimensional Computed Tomography to 4-Dimensional Cone-Beam Computed Tomography Deformable Image Registration for Lung Cancer Adaptive Radiation Therapy." Int. J. Radiat. Oncol. Biol. Phys. 86, 372–9*

http://www.cancerimagingarchive.net/ or http://doi.org/10.7937/K9/TCIA.2016.ELN8YGLE.

- *Note: Additional datasets available in TCIA, with search fields.*

18. H&N datasets (Pukala *et al* http://dx.doi.org/10.1118/1.4823467)

- Set of 10 head & neck pairs (without contour or landmarks)
- The ground-truth DVF is not part of open source.
- The previous available DVF analysis is no longer available since Jan 2018, as it's now part of purchasable items in ImSimQA DIR.
- *Jason Pukala J, Meeks S, Staton R, Bova F, Mañon R, Langen K*. 2013. A virtual phantom library for the quantification of deformable image registration uncertainties in patients with cancers of the head and neck. Med Phys 40(11) 111703

https://sites.google.com/site/dirphantoms/virtual-phantom-download

19. TG 132 Datasets: Use of image registration and fusion algorithms and techniques in radiotherapy (https://www.aapm.org/pubs/reports/report132.asp) based on AAPM TG132 report
https://aapm.org/pubs/reports/detail.asp?docid=164
